# Histogenesis of the Oesophagus of Guinea Fowl (*Numida meleagris*) at Prehatch and Posthatch

**DOI:** 10.1155/2016/9827956

**Published:** 2016-12-08

**Authors:** Innocent Jonah Gosomji, Sulaiman Olawoye Salami, James Oliver Nzalak, Muhammed Umar Kawu, Emmanuel Vandi Tizhe, Yilzem George Gurumyen, Edward Christopher Dung

**Affiliations:** ^1^Department of Veterinary Anatomy, University of Jos, Plateau State, Nigeria; ^2^Department of Veterinary Anatomy, University of Ilorin, Kwara State, Nigeria; ^3^Department of Veterinary Anatomy, Ahmadu Bello University, Zaria, Kaduna State, Nigeria; ^4^Department of Veterinary Physiology, Ahmadu Bello University, Zaria, Kaduna State, Nigeria; ^5^Department of Veterinary Microbiology and Pathology, University of Jos, Plateau State, Nigeria; ^6^Department of Veterinary Physiology, University of Jos, Plateau State, Nigeria

## Abstract

The histogenesis of the primordial oesophagus was studied to determine the period in which the tunics of the oesophagus developed and became functional in the helmeted guinea fowl (*Numida meleagris*). Eighteen embryos and nine keets were studied at prehatch and posthatch, respectively. Simple columnar epithelium surrounded by mesenchymal cells was obvious at the 8th day of embryonic development. By the 19th day of embryonic development, the four tunics, tunica mucosa, submucosa, tunica muscularis, and tunica adventitia/serosa, were beginning to differentiate from the mesenchymal cells and also the primordial oesophageal glands appeared as clusters of cells that invaginate from the epithelium. By the 27th day, the tunics were clearly differentiated and the primordial glands were fully developed as evident with positive reaction to Periodic Acid Schiff (PAS). The tunics of the muscularis were not well developed till at posthatch. This study therefore concludes that the primordial oesophagus is active at the late incubation due to mucin secretion by mucous glands but fully functional at posthatch since the tunica muscularis is completely developed at posthatch.

## 1. Introduction

The digestive process of food animal must be functional to ensure safety and healthiness of the animal product consumed by humans [1]. This is realized by the structures of the digestive system which provides an environment for the physical and chemical reduction in the size and molecular complexity of food and then absorbs the end products [2].

For the ingested feed to reach the stomach for digestion, it has to first pass through the oesophagus which is thin walled and highly dilatable [3] due to the inability of the birds to break down food orally [4]. It consists of two parts in birds, the cervical and thoracic while in ruminant, the abdominal oesophagus makes the third oesophageal segments [5–7].

The primordial oesophagus of the guinea fowl appeared on the 8th day of incubation and separated into the cervical and thoracic oesophagus on the 11th day of incubation [5]. It extends from the tracheal groove to the fusiform dilation of the primordial stomach [8].

This work was aimed at describing the histological and histochemical features of the primordial oesophagus and also to determine the period in which the oesophagus is functional in the helmeted guinea fowl (*Numida meleagris*).

## 2. Materials and Methods

Eighteen embryos of helmeted guinea fowl (*Numida meleagris*) at incubation days 8, 11, 13, 19, 24, and 27 were utilized for prehatch studies while nine keets at days 1, 5, and 8 were utilized for posthatch studies. Three per day were collected from Dhenab hatchery, Angul-D, Jos, Plateau State. The incubation was maintained at a temperature and relative humidity of 37.7°C and 60–70%, respectively. The removal of the embryos from the eggs was done according to the method described by Salami [9]. The shells of the eggs were cracked at the broad end with forceps to create an opening of approximately one inch in diameter. The outer and inner shell membranes were removed using small pointed-end scissors. The cracked eggs were preserved in 10% formalin. The embryos at days 19, 24, and 27 days and also at posthatch were euthanized using 0.1 mL of phenobarbitone (200 mg/mL) via jugular vein [10].

Samples taken were immediately fixed in 10% formalin. The fixed tissues were dehydrated through a series of graded alcohol (70%, 80%, 90%, 95%, and 100%). They were cleared in xylene and embedded in molten paraffin wax. Transverse sections of 5 *μ* thick were cut from the embedded tissue using disposable microtome knives. These sections were mounted on clean and grease free slides and were also stained at room temperature using Haematoxylin and Eosin (H and E) to analyze tissue structure and Periodic Acid Schiff (PAS), a histochemical technique to analyze the activity of glycosaminoglycans [11].

The prepared slides were viewed under a binocular light microscope with a digital camera (Sony, 16 megapixels) fitted to the eye piece of the binocular light microscope and adjusted at desired magnification and photomicrographs of the histological features taken.

## 3. Results

**Figure 1:**
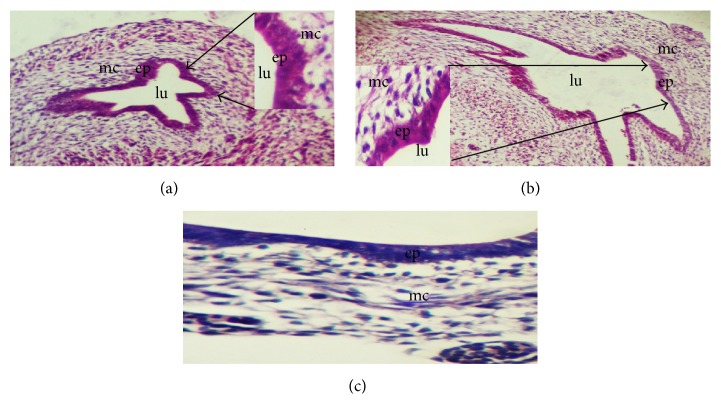
Sections of the primordial oesophagus on the 8th (a) and 11th (b) days of development showing the two layers, the epithelial and the mesenchymal layers. On the 8th day the epithelial lining was simple columnar while on the 11th day there was both simple columnar and stratified squamous epithelium. H and E ×100 (a), ×100 (b). (c) shows weak reaction of the epithelium at the 11th day of development. PAS ×400.

**Figure 2 fig2:**
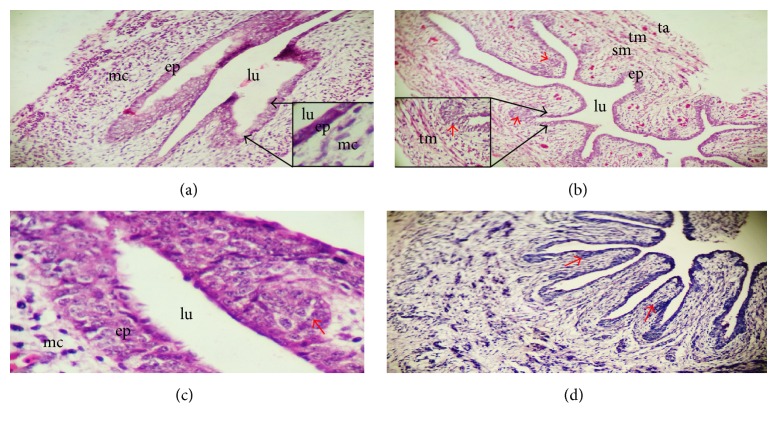
Sections of the primordial oesophagus on the 13th (a) and 19th (b and c) days of development. The 13th day shows the development of the longitudinal folds which project into the lumen (lu). The 19th day shows the formation of the four tunics, the tunica mucosa lined by stratified squamous epithelium (ep), submucosa (sm), tunica muscularis (tm), and tunica adventitia (ta). H and E ×100 (a, b) and ×400 (c). The primordial oesophageal gland (red arrows) appeared in clusters in the tunica mucosa. (d) shows weak reaction of oesophageal glands (red arrow) on 19th day of development. PAS ×100.

**Figure 3 fig3:**
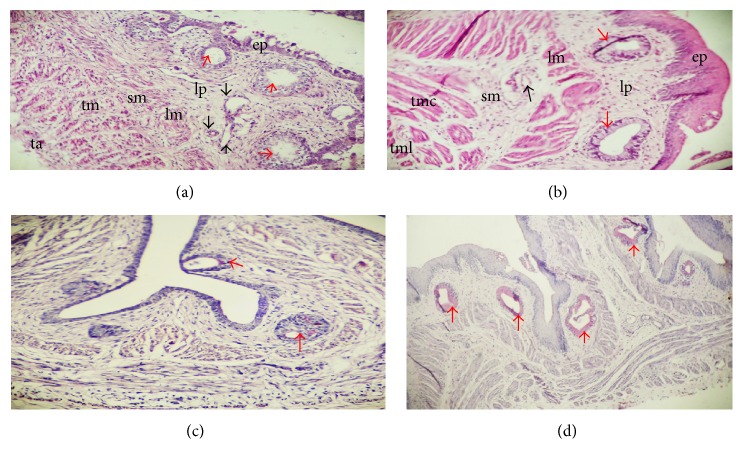
Sections of the primordial oesophagus on the 24th (a) and 27th (b) days of development; the 24th day of development shows primordial oesophageal glands (red arrow) with single and stratified layers and also thick tunica muscularis (tm); on the 27th day, primordial oesophageal glands showed single layer of cuboidal cells, lamina propria (lp) clearly separated from lamina muscularis (lm), submucosa (sm), blood vessel (black arrow), inner circular muscle (tmc), and outer longitudinal muscle (tml). (c) and (d) show histochemical characteristics of primordial oesophagus on 24th and 27th days of development, respectively; 24th day shows weak reaction of oesophageal glands (red arrows) while 27th day shows positive oesophageal glands reaction. PAS ×100 (c), ×40 (d).

**Figure 4 fig4:**
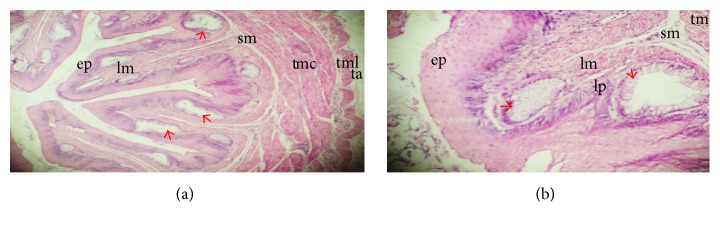
Sections of oesophagus on days 1 and 8 posthatch showing matured epithelium (ep), lamina propria (lp), oesophageal glands (red arrow) lamina muscularis (lm), submucosa (sm), inner circular layer of tunica muscularis (tmc), outer longitudinal tunica muscularis (tml), and tunica adventitia (ta).

**Figure 5:**
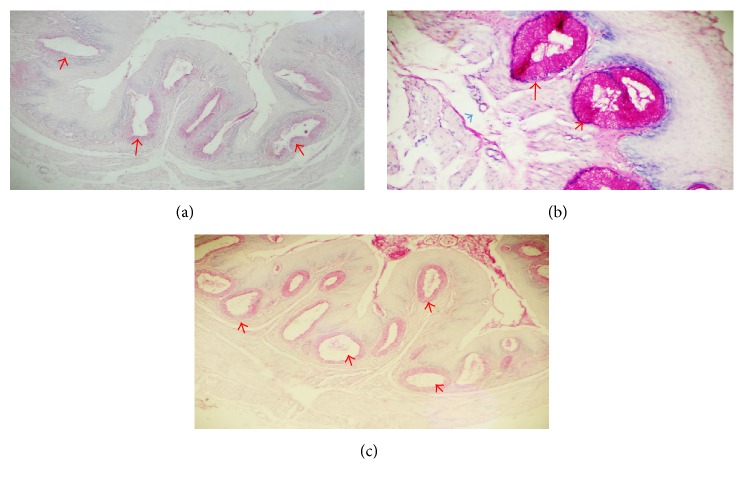
Histochemical characteristics of the cervical oesophagus, crop and thoracic oesophagus on days 1, 5, and 8, respectively, posthatch. The oesophageal glands (red arrows) indicate strong positive reaction. PAS ×40 (a), ×100 (b), and ×40 (c).

The primordial oesophagus of the helmeted guinea fowl showed progressive development of the four layers of a typical oesophagus: tunica mucosa, submucosa, tunica muscularis, and tunica adventitia/serosa. The tunica mucosa contained numerous longitudinal folds lined by stratified squamous nonkeratinized epithelium with numerous oesophageal glands in the lamina propria and few layers of smooth muscles making the muscularis mucosa. The submucosal layer contains blood vessels, lymph vessels, and plexuses. The tunica muscularis contained striated muscular fibers. Outer tunica adventitia was a thin layer of connective tissue surrounded by mesothelium (Figures 1 [Fig fig2] [Fig fig3] [Fig fig4]–5).

The cervical oesophagus, crop, and thoracic oesophagus were observed to have similar histological development at both prehatch and posthatch. By the 8th day of development, two layers were formed: the mucosal layer lined by simple columnar epithelium surrounded by undifferentiated cells also known as mesenchymal cells (Figure 1(a)). The lumen appeared star shaped and wider by the 11th day of embryonic development (Figure 1(b)) and also the epithelial lining at this stage reacted weakly to PAS (Figure 1(c)). By the 13th day of the embryo, the mucosa projects into the lumen forming longitudinal folds (Figure 2(a)). These folds maintained their lining of the epithelium. By the 19th day, the epithelial cells at the basal pole began to proliferate to form clusters of primordial oesophageal glands while towards the surfaces the epithelium had gradually changed to stratified squamous epithelium (Figures 2(b) and 2(c)). Still on the 19th day, the undifferentiated cells began differentiating forming submucosa, strands of striated muscles of the tunica muscularis, and adventitia/serosa. The epithelial lining reacted weakly to PAS (Figure 2(d)). By the 24th day of the embryo, a wide lumen has been created in each of the primordial oesophageal glands displacing the cells from the center, thereby giving it a spherical appearance (Figure 3(a)). Some of these primordial oesophageal glands were simple columnar while others appeared stratified (Figure 3(a)). The epithelium and primordial glands reaction was weak to PAS (Figure 3(c)). By 27th day, the layers are matured with the mucosal layer having nonkeratinized stratified squamous epithelium, lamina propria having all the primordial glands lumen lined by simple cuboidal epithelium giving the characteristics of mucosal glands, and well-developed lamina muscularis; the submucosa was clearly separated from the other layers; the tunica muscularis had the inner circular and outer longitudinal muscles; adventitia/serosa are also clearly separated from the tunica muscularis (Figure 3(b)). The primordial oesophageal glands reacted positively to PAS (Figure 3(d)). This is an indication that the glands are highly active on the 27th day of embryonic development.

At posthatch, the tunics are clearly separated with the following features: the tunica mucosa constituting the nonkeratinized squamous epithelium, pockets of oesophageal glands extending towards the epithelium at the lamina propria, and the lamina muscularis containing bands of muscles; thin submucosa containing connective tissues; tunica muscularis with the inner circular and outer longitudinal muscles; and outer covering of thin loose connective tissue, the tunica adventitia/serosa (Figures 4(a) and 4(b)). The glands demostrated the presence of mucin on staining with PAS which is an evidence of secretory activities of the oesophagus (Figures 5(a), 5(b), and 5(c)).

## 4. Discussion

The oesophageal histogenesis of helmeted guinea fowl (*Numida meleagris*) appears to be similar to most avian species. The changes observed in the primordial oesophageal mucosa from simple columnar cells to stratified squamous epithelium corroborate the findings in mouse [12]. This varies slightly with the observation of Schumacher [13] that observed earlier pseudostratified epithelium, before the columnar, and then the stratified squamous epithelium at the later stage of development.

Underneath the epithelium is the lamina propria which originates from the densely appeared mesenchyme [14]. It is a network of connective tissue with sparse distribution of oesophageal glands. The appearance of primordial oesophageal glands on the 19th day of embryonic development of guinea fowl was similar to that of chick embryo on the 16th day of embryonic development [15]. The less active secretory cells of the oesophageal glands on the 19th to the 24th days of embryonic development were meant to compensate mucosal lining since the glands have not been fully developed to mucous glands. The positive reaction of the primordial oesophageal glands to PAS at the 27th day of embryonic development indicates that the glands secrete mucin, an indicator of activity of the glands. These observations agree with the findings of Ventura et al. [16].

The tiny submucosal layer of the helmeted guinea fowl exists between muscularis mucosae and tunica muscularis where blood vessels, nerves, and other connective tissues are found [17].

There was no clear developmental difference between inner circular and outer longitudinal muscles of the tunica muscularis and the muscles are not fully developed at the entire period of prehatch, although Ivey and Edgar [14] observed the different muscles at embryonic development in the chick and guinea fowl. There was no specification of the species of guinea fowl which might be the reason for the difference in development. They also reported that in pigeon, the muscles were developed in an unusual manner with the longitudinal layer either poorly developed or absent.

The nonkeratinization of the stratified squamous epithelium of the oesophagus of guinea fowl at posthatch in this study appeared to be similar to the findings in chickens [18], geese [19], and quails [20]. Just beneath the epithelium is the lamina propria, a layer of connective tissue containing mucosal glands extending towards the epithelium. These glands are only found in the lamina propria to corroborate the findings in fowl [21]. Longitudinal muscularis mucosa an extension of muscle lying close to the mucosal glands might be responsible for putting pressure on the glands resulting in contraction to release mucin [22]. Thin submucosal layer has been reported in most avian species [23] but missing in grey-backed shrike [22], while the tunica muscularis appeared broad with inner circular and outer longitudinal external muscle layer in avian species [18, 19, 24–26]; these appeared similar to the findings of this study.

## 5. Conclusion

The primordial oesophagus was active on the 27th day of development due to mucin secretion by the oesophageal glands. At posthatch, the inner circular and outer longitudinal muscles became fully developed.
